# Attachment-based psychosocial programme for under-privileged school children with adverse life experiences in Istanbul, Turkey

**DOI:** 10.1186/s40359-022-00938-x

**Published:** 2022-10-08

**Authors:** Nasir Warfa, Özden Bademci, Şahin Karasar, Robert White

**Affiliations:** 1 Maltepe University Research and Application Centre for Street Children, Maltepe University, Marmara Eğitim Köyü, 34857 Maltepe, Istanbul, Turkey; 2 Division of Psychology, Sociology, and Education, School Effectiveness and Improvement, Queen Margaret University, Edinburgh, UK

**Keywords:** Attachment Theory, Psychosocial Models, Istanbul School Children

## Abstract

Children experiencing educational neglect are likely to experience the harm associated with adverse life experiences and a range of emotional and psychological challenges. Using attachment theory and psychosocial frameworks, we devised and implemented an intervention designed to ameliorate the deleterious effects of challenging behaviours in an elementary school situated in a deprived neighbourhood of Istanbul, Turkey. For a period of eight months, 160 pupils received a psychosocial intervention once a week. Children’s patterns of behaviour, emotions, movements, interactivity, socialisation and interpersonal communications were observed during this period. Core activities of the intervention included library visits, reading, writing and listening games, maths games, drawing, animal animation, leaf making, ball games, parachuting games, colouring, hula-hoop and driver-car role plays. At the end of the intervention, one group of children made significant improvements in behavioural changes while no improvements were observed for another group. Although further research is required to generalize beyond the reference group, the findings suggest that a robust collaboration between inter-agency community partnership and universities can play a crucial role in responding to the needs of marginalized children with psychological and emotional problems.

## Introduction

Education is a key driver of social and economic development and has a profound effect on population health. Although Turkey is a middle-income country, the number of children facing school exclusion or leaving school at early age with no qualifications is significantly higher than other EU members [13]. The UN developed a *human-rights-based approach to education for all* [25]. The human-rights approach to ‘education for all’ provides a holistic framework that confirms the right to inclusive quality education be guaranteed by all member state for all people [25]. Nevertheless, faced with limited resources, many schools in Turkey struggle with the provision of inclusive education for children living in poor neighbourhoods [5]. Children attending such deprived schools are more likely to experience life difficulties, precarity, and educational neglect. We adopted the following definition of educational neglect: “*a situation in which a caregiver knowingly allows chronic truancy (five or more days a month), fails to enrol child into school or repeatedly keeps child at home* ([26]: 52). The long-term outcome of educational neglect often results in early school leavers working in poorly regulated and unsafe environment [4, 6]. Both qualitative and quantitative data highlight the social and psychological repercussions educational neglect have for children. We carried out a recent study [2] with four European countries (Bulgaria, Italy, Malta, Romania) and Turkey which showed that people with educational neglect were almost three times more likely to be neither in employment nor in education and training. The study showed that early school leavers with low or no education were more likely to suffer from poverty and social exclusion. They were also more likely to raise a generation of children who were not motivated to learn or given the opportunity to attend school. Another study [4] with street children in Turkey found that poverty and generational continuity of low education pushed a significant number of children into illegal child labour. Previous studies found that children at risk of educational neglect were more likely to engage in risky behaviours and criminal activities while trying to meet essential needs such as food [4, 10]. In short, from Turkey and elsewhere, children exposed to educational marginality go on to experience abject poverty and difficult life experiences throughout the life circle.

Moreover, children experiencing educational neglect have a higher exposure to traumatic abuses and are more likely to experience emotional and behavioural problems [18]. Marginalized children are more likely to experience physical health problems, psychological maltreatment, poor self-esteem and adverse childhood dysfunction. Research by Metzler et al. [18] analysed population data from 10 states (USA) to examine the associations between early adversity, adult socioeconomic status (education, employment and income) and health across the life course. They found that people with high scores of adverse childhood experiences were less likely to complete school and more likely to live in households below the federal poverty levels. They discussed these findings in relation to structural policies and processes designed to break the intergenerational continuity of child abuse and educational neglect.

Felitti et al. [14] examined the relationship between exposure to childhood (emotional, physical, and sexual) abuse, health risk behaviour and adult household dysfunctions. They found that individuals who had experienced four or more categories of adverse childhood events had four to 12-fold increased health risks for alcoholism, drug abuse, depression, and suicide attempt. They reported that multiple categories of adverse childhood experiences were strongly interrelated and children with these multiple categories of abusive exposures were likely to have multiple health risk factors later in life. In a nutshell, the global literature suggests a strong association between educational neglect, childhood adversities, socioeconomic disadvantages, household dysfunction and poor health behaviour.

### Attachment classification and psychosocial models

The attachment theory is a major constituent of the psychological understanding of emotional bonds, relationships, and development. It is frequently and quite extensively written and citied in the literature which is why we will not review it here in detail. Briefly, the theory illustrates the importance of providing a secure child–adult attachment system where children learn how to regulate emotions such as fear under the supervision of adult caregivers ([8]; citied in Warfa et al., [27]). A safe and secure fear regulation mechanism enables the child to cope with stressful emotions and difficult psychological situations, and thus found to be crucial for later emotional maturity and better life outcomes in adulthood. Bowlby developed two models of attachment style: secure and insecure. Examples of the insecure attachment construct include anxious, ambiguous, avoidance and disorganised behaviour. The type of child-parent relationship experienced during the early formative years influences the style of attachment system a child develops. Secure attachment is formed through the provision of responsive and caring relationship between the caregiver and the child.

Ainsworth [1] discusses the concept of “attachment bonding and relational stance” where the child relies on a “wiser and older” adult for a stable loving relationship over time. If the child receives limited protection and love during the early developmental stages, the style of bonding and attachment becomes ambivalent and disorganized. In other words, caregiver’s ‘maintenance’ of emotional availability, emotional sensitivity, relational bonding and empathic resonance from early years protects the psychological and emotional wellbeing of later adult life [3]. These are the core elements of a secure attachment system. Insecure attachment emerges from an insensitive or unresponsive child-parent/child-caregiver relationship [8]. The failure to develop and maintain a supportive relationship and positive attachment interactions leads to the rise of problematic types of behaviour. An insecure attachment behaviour that is destructive to mental health and the ability of the child to respond to difficult psychological experiences throughout life. While secure attachment-bonding is established as a protective factor for long-term psychological well-being, inconsistent attachment styles are linked to low self-esteem, bad temperament, child developmental problems, relationship problems, adjustment difficulties, poor educational attainment and poor quality of adult life [3, 6].

### Psychosocial models

Unlike the attachment theory, the psychosocial model is not informed by one single theory. Instead, it is an approach which endeavours to address “the totality of a person” [20, 21]. Although the application of the psychosocial approach is far wider than the attachment theory, there is not a clear definition of the concept. One of the most coherent explanations is offered by Papadopoulos [20, 21] and Papadopoulos and Gionakis [19] who clarify that a comprehensive psychosocial approach includes three inter-related dimensions: intrapsychic, interpersonal and socio-political. The intrapsychic processes refer to the inner experiences of a person, for example, feelings, fears, hopes and wishes. The interpersonal dimension addresses the interactions which take place between individuals, and the socio-political pillar takes into consideration not just psychological processes and interpersonal relations but also the wider political and socio-economic factors that can influence health and well-being.

Furthermore, in the process of developing an interdisciplinary study, we turned to Gonzalez et al. [16] review which addressed four psychosocial conceptual frameworks. These are: self-efficacy, coping, learned helplessness, and social support. For instance, self-efficacy activates one’s belief system (self-belief), motivation and psychological abilities to achieve set goals or overcome life adversities. Role modelling is a good demonstration of self-efficacy where people who managed to turn around difficult life events inspire others experiencing similar situations to achieve positive behavioural change. The psychosocial theory of coping is also useful here. Coping is a cognitive and psychological strategy which is invoked in the face of enduring circumstances. Having the ability to bounce back and having good relationship with others are both an essential part of a resilience strategy, a psychosocial resilience strategy that can be strengthened and nourished through supportive early schooling. The psychosocial intervention we are reporting in this paper are informed and guided by the attachment theory and the above-noted psychosocial models. The aim was to formulate an attachment and psychosocial informed programme for vulnerable school children with multiple experiences of educational neglect, community deprivation and difficult childhood life events.

## Method and methodology

We identified several attachment-based classifications and psychosocial models that are relevant to steer this work. We used Attachment Aware Schools (AAS) as a general framework for delivering and implementing the psychosocial programme. The Attachment Aware Schools framework helps to nurture healthy relationship which is found to be beneficial to the socioemotional development and learning process of children with insecure attachment experiences [24]. From the Attachment Aware Schools framework, children’s socioemotional distress is regulated through a responsive attachment system and psychosocial coaching in a supervised educational context. We also implemented the psychosocial programme within the methodological frameworks of Social Action Research [12]. Social action research partly emerged in response to concerns of the time it takes to conduct traditional research with limited funding as well as the time it takes to implement and disseminate research outcomes. Social action research is collaborative in nature for its success depends on several stakeholders to carry out the field work. This approach is based on a constructivist principle which acknowledges the creation and dissemination of evidence-based knowledge through human interactions. McWilliam et al. [17] discusses how psychosocial interventions can be implemented through a social interaction process illustrating, for example, how the documentation and facilitation of the social interaction process itself is a critical part of implementation science. In the process of setting up this psychosocial school programme, we collaborated with core stakeholders including school management, teachers, school support staff, administrators, community groups, local district municipality, student mentors and academics from different university departments and faculties.

### Participants

We devised an innovative psychosocial programme where 25 psychology student mentors and their professors provided weekly psychosocial support to primary school children over a period of eight months. The psychosocial intervention consisted of three main pillars. In the first two pillars, the psychology student mentors were supervised to provide one-to-one and in group psychological support to strengthen children’s emotional development (intrapsychic element). Also, the in-group and one to one meetings and programme activities were designed to promote socialisation and positive social interactions within a secure educational setting (interpersonal pillar). Papadopoulos’ definition of what psychosocial is included socio-political elements (the third pillar). As we noted above, the implementation of this psychosocial intervention was made possible by collaborating with a wide range of stakeholders from different sectors and community groups. We will address the wider political and socio-economic aspect of the intervention in the discussion section.

The primary school in which the intervention took place is in a deprived part of Istanbul. There were concerns that children attending the school were exhibiting a range of behavioural and emotional problems akin to insecure attachment system. Community members with worries about high level of absenteeism, bullying and disruptive behavioural problems among the school children approached academic colleagues for support. Following several meetings held with the school’s management, parents and local education municipality, we set up an innovative programme where university psychology student-mentors were supervised to provide weekly psychosocial support to the primary school children (Also see [3]).

The psychology student mentors received comprehensive training and weekly supervision to deliver the psychosocial intervention to four classes of year-one pupils (seven years old). We focused on year-one pupils partly because we had limited capacity and limited resources to implement the psychosocial programme across the school. After a thorough consultation and meetings with core stakeholders, we selected the year-one group for the intervention since they were identified as the group that were most likely to benefit from the intervention [3]. As well as receiving supervision three times a week during the entire duration of the programme the psychology students were getting onsite programme supervision and ongoing monitoring. Lastly but not least, the university student mentors had gone through psychomotricity training from a psychomotricity body psychotherapist. Psychomotricity views the individual as a whole and in relation to their external environment and follows the interactions between the motor and psychological components. This helps the individual to adapt in a flexible and harmonious way to the environment around them [7]. Psychomotricity aids the learning process and improves memory, attention and concentration [11]. Psychomotricity brings together the physical body and psychological aspects of the self in a holistic way and this makes the person to be more aware (mentally and physically) of their actions and the environment within which they are interacting with others [7]. We used psychomotricity to help the student mentors and school children to deliver and receive the psychosocial intervention within a safe and respectful educational environment.

### Procedures

There were 4 classes of year one. Each class had a capacity for 40 pupils (160 pupils in total), although approximately 20 pupils (almost half of year one pupils) would be present in the class in any given week. For a period of eight months, every Friday of the week (seven hours in total per week), the university student mentors would interact with the school children in the four classes to observe the children’s patterns of behaviour, emotions, movements, interactivity, socialisation and interpersonal communications. Once the initial observations were completed, the psychology students and class teachers would identify pupils with the most emotional and behavioural needs from the four classes. Pupils in each class (Class A, Class B, Class C and Class D) were divided into smaller sub-groups. For example, Class A, group 1, Class A group 2, Class A, group 3 and Class A, group 4, depending on the number of pupils present in the class each week. Each group (groups 1, 2, 3 and 4) consisted of both children with and without emotional and behavioural problems. Those with repeated problematic behavioural patterns and emotional needs (up to five students from a class of 20 pupils) were selected for focused one-to-one support, receiving the same psychosocial intervention as detailed above.

The weekly psychosocial programme was carried out under five key stages: observation, action, impact, challenges and reflections. In stage 1, university student mentors would observe and evaluate the children’s behaviour, emotions, movements, attendance, interactivity, socialisation and interpersonal relations in groups and on one-to-one basis. In the second stage, they implemented psychosocial actions that were designed to address the emotional and behavioural needs identified in stage one. The intervention included the following learning activities: library visits, reading, writing and listening games, painting games, maths games, handwashing games, drawing, animal animation, calendar making, leaf making, puzzle games, garden games, ball games, parachuting games, colouring and hula-hoop and driver-car role plays.

Every activity was intended to lead to an improvement in emotional and behavioural wellbeing of the school children. The activities were also intended to improve social and interpersonal skills. For instance, the car-driving role play is a game that is aimed to stimulate the pupils’ logical thinking and conscientiousness in terms of adhering to and respecting commonly agreed rules. Other activities (such as garden and ball games) were intended to maximise positive social interactions, another key component of the psychosocial programme. In stages 3 and 4, the impact of the actions taken in stage two (positive or negative) were assessed and documented. What worked or did not work and for whom was evaluated and reflected on. New action plans were devised to address challenging behaviours that remained unresolved. Stage 5 provided the university student mentors a reflective space through which they carried out weekly reviews and discussions of the impact of the psychosocial work on the school children as well as how this work impacted on their professional and personal development.

Ethical approval was obtained from Maltepe University Ethics Committee. Permissions to work with children in the school were also given by the District National Educational Directorate, District Governor, and the Local Municipality responsible for the administration of the district schools. Informed consent for children to participate in the study was obtained from parents/cares. We anonymized the names of the children used in the manuscript.

### Analysis

Each time the psychology student mentors delivered the psychosocial intervention, they were required to write down both individual and group observations under supervision. Over a period of eight months, the student mentors were directed to report on any behavioural changes they observed during the programme activities. Over the same period, we carried out weekly discussions to review if the psychosocial programme had any impact. Supervised observations from individual student mentors and group sessions were then compiled into four large reports. We carried out four-months of intense data analysis into written reports compiled from the eight-months-long psychosocial programme.

We focused on data related to the constructs of violence, bullying, disruptive behaviour absenteeism, attachment behaviour, attachment relationships and behaviour changes. Data were organised and analysed under the categories of Observation, Action, Impact, Challenge and Reflection. We used both documentary and thematic analysis. Documentary analysis is a core part of both qualitative and quantitative methods and has been used to analyse written narratives and reports for over a century. Scientific Documents are described *as social facts which are produced, shared, and used in socially organised ways* (Coffey, 1997, cited in Bowen, 2017). The thematic analysis method was used to identify, code and recode key themes from the four compiled reports containing the weekly observations. From this perspective, evidence-based knowledge is generated from and through the weekly psychosocial interactions and observations. The thematic analysis method provides simple but a realist thematic framework that is flexible and accessible. Braun and Clarke [9] suggest six steps that are necessary for thematic analysis. The first step alludes to the state of becoming familiar with the data by reading and rereading it. In the second step, relevant themes are searched and coded systematically. Core themes of interests are then mapped and categorised in step 3. Step 4 involves the reviews of the main relevant themes. Step 5 involves ongoing analysis and tuning of the mapped and categorised themes. This is followed by the final step (step 6) where examples of selected extracts and quotes are analysed and then discussed. To add a new analytic layer of rich data interpretation, we applied the Interpretive Phenomenological Analysis [23]. This allowed us to gain additional in-depth and analytic interpretations of the meaning and context attached to the descriptive psychosocial data we are reporting here. We anonymised the identity of the children. No child is named in the manuscript.

## Results

From the weekly individual and group observations, we were able to document any behavioural changes or emotional improvements the children made in response to the psychosocial programme activities. For example, from the weekly interpersonal communications and from activities such as library visits, reading games, writing games, listening games, maths games, social games, animation, parachuting games, ball games and so on. Tables 1 and 2, summarise the findings of all four year-one classes (Class 1, groups ABCD; Class 2, groups ABCD; Class 3, groups ABCD and Class 4, groups ABCD). The data presented in Table 1 emerged from individual and group observations. There is a footnote at the end of the Table if the texts are coming from group or individual mentor observations. We found little differences between the individual and group observations. To avoid duplications, we present examples and quotes which relate to diverse individual child experiences. Table 2 has information on absenteeism. From all year-one classes, violence, bullying, provocation, absenteeism, emotional and psychological problems were identified as reoccurring themes. Table 1 provides specific examples of the emotional and behavioural improvements children made following the introduction of the attachment-based psychosocial programme.

**Table 1:** Bullying, violence and behavioural problems

	Improvements over time: some examples
MT is too aggressive towards friends, and always fights Classmates were consistently hitting ERN on the head but said nothing CU is clingy and does not express herself, she was yelling to get our attention SI is restless and needs individual care MD had aggressive behaviour towards others UZ was restless and distracting YA was shy and her mood low AZ showed impatient, aggressive and violent behaviour TZ is generally silent and has limited interactions EH was very aggressive and constantly fights with friends RI aggressive and uncooperative CA was very aggressive and angry MZ constantly runs around in the class, is aggressive and swears SO was beaten by two other students yesterday UTM did not come to school for too long and distracts others when he comes PA is shy and has avoidant attitude IY is unhappy and always emphasizes her illness ERN is easily distractible ST came to school only twice in this semester YZ is hyperactive, angry and aggressive CX was very violent, not respecting class rules, or willing to cooperate with other children CG was violent, overactive, rebellious, uncooperative CH is clingy, a little bit aggressive, uncooperative and has concentration problems CD is always absent and lacks focus CE just argues, swears, fights and has anger issues CS was violent, overactive and rebellious LA is challenging if something had not gone in his way YB would take things without permission and refuse to share	NY is more focused and motivated KI is more confident and felt free to express through drawing CU felt supported and is respectful. She enjoys and expresses through painting LB started to learn how to draw and it improved his social skills MI is less shy and in a better mood, participates more PA’s confidence and willingness to engage in the class activities improved every single week LO was no longer clingy but affectionate and felt comfortable through the painting game SN is more cheerful and less aggressive. I never expected him to make such a huge progress PE is far less shy, very social and has hesitant attitudes disappeared MO enjoyed playing the games, felt supported and this encouraged to do the class work joyfully NR’s relationships with friends and others are better and is more active lately ZZ improved communication and social skills. He/she is more enthusiastic GZ is less shy and communicates with friends easily SI is less violent, more cooperative, respects the rules and seeks less attention MD was very aggressive at the beginning but is not aggressive with friends now YZ is more focused and inspired during the painting game UZ is less restless, less disruptive, more focused and calmer SO listens more, pays more attention and relationship with friends became stronger MZ could not focus or listen at the beginning but now pays attention TZ hardly communicate with anyone at the beginning, communicates with everyone now YA had poor self-confidence and felt inadequate. Self-confidence improved through the game activities CZ was very violent at the beginning but is calm towards friends now CS started to concentrate better on daily basis RI got a way better in the coming weeks LA got better every week BK is a former shy kid and enjoys the school more YB learned how to share and would seek permission before taking things

CX, CG, CH, CD, CE (group observations)

**Table 2:** Participant absenteeism recorded for duration of the intervention

CU was absent for a few weeks	IY absenteeism is regular
SU was absent for several weeks	UTM was absent for a few weeks
KI was absent for 11 weeks	BH was not in school for the first term
MI was absent for 11 weeks	EZ rarely comes to school
PE was absent for the past weeks	ER is always absent
RR was absent for 10 weeks	MZ was absent for 5 weeks
NA was absent for a few weeks	SZ was absent for 5 weeks
MO was absent for 6 weeks	YZ was absent for 3 weeks
MI was absent for 11 weeks	SE was absent for 4 weeks
CA was absent for 4 weeks	SY was absent for 6 weeks
AN was absent for 5 weeks	GD was absent for 4 weeks
EFR was absent for 9 weeks	ST came to school only twice in this term
MY started school 3 months later	AZ was absent for 10 weeks

### Children with improved emotional state

During the initial observations, we gained insights into the types of emotional and behavioural problems the school children exhibited. We then implemented the intense weekly psychosocial learning activities to create an attachment-based learning environment. At the end of the eight-month period, one group of children made significant improvements while no notable changes were observed in another group of children. We will start with the discussing the outcomes of the children displaying improvement within the categories noted in the intervention:

MD was very aggressive and talked dirty. Now, she is very calm and respectful. She is not showing aggressive behaviour and talks politely with friends.

RI was aggressive and uncooperative. On one Friday, she was yelling and would not listen or participate in activities. She got a way better in the coming weeks. She started to be open more and became cheerful.

CZ was aggressive and used to fight with friends. He gradually learned to stay calm.

CS was violent, distractive, overactive and rebellious. After a few months, the rebellious attitudes started to decline. He started to concentrate better on daily tasks… A kid punched him and he just walked away, there has to be a miracle! There was really a positive impact on him.

The above quotes illustrate that the psychosocial programme motivated children to moderate their disruptive behaviour and negative attitudes. Over time, they became calmer, cheerful, polite and less aggressive. Adverse childhood experiences are known to slow the learning process and impair educational attainment. From the attachment theory, children develop emotional and behavioural problems when they are exposed to insecure attachment behaviour. These emotional and behavioural problems disrupt the child’s educational achievement. Since children with insecure attachment experiences struggle to complete school education, we provided a supportive psychosocial environment where crucial social and interpersonal relationship skills were prioritized:

At the beginning, SI was violent, not respecting rules and was not willing to cooperate. I can see she does not ask for special care as much as before. She is cooperative and improved social skills.

SO had difficulty in focusing when others are speaking. Over time, this problem greatly diminished, which strengthened friendship skills to a considerable extent. We had one missing brush during an event when he volunteered to share his own.

ST needed to be listened to or seen by someone. There was a big change in her behaviour, and relationships with others, and don’t fight with friends anymore.

BB was always fighting with friends and the boys in the class complained about this. He would not sit down in the lesson, and constantly walked around, talking with friends. Over time, he became more compatible with the group.

These observations indicate how the provision of a caring and trusting learning space enabled children to be cooperative and become friendlier. They showed less disruptive behaviour, less emotional problems, and more respect. In our previous study [29] we reported the beneficial impacts of a pro-social learning programme on building a positive school climate and inclusive character education. Six universally appreciated and valued personality traits were crucial for building such inclusive schools of character. These were respectability, responsibility, trustworthiness, honesty, fairness, and caring. To some extent, the attachment-based psychosocial programme contributed to the building of a positive educational character:

YA is a child who looks small as a physical appearance. She lacks self-confidence and inadequate because of physical appearance. She had a lot of fun when she joined the games, which improved her self-confidence.

GZ presented us with different challenges every week. This term, she follows the game rules, communicate more easily. She does not seek attention and waits for her turn during the games.

LA would be challenging if something had not gone in his way. He got better every week. He is now very understandable because he understands he cannot be at his best all the time.

BK is one of the “former” shy kids. He enjoys school more and made nice improvements.

CY has come a long way. At the beginning, she was clingy, a little bit aggressive, had difficulties with concentration. As time passed, she kept getting better and better. She is more confident, friendlier and excited. She just needed support, affection and comfort, which motivated her.

The provision of an attachment-based psychosocial programme space contributed to improved child behaviour, improved emotional wellbeing and enjoyable school environment. This is also consistent with the Attachment Awareness Schools model (AAS: [22]), where *setting specific training packages and fostering of universal and specialised attachment-based strategies* are cited as key elements.

The Psychosocial programme strengthened children’s emotional and psychological well-being as they became less clingy and less attention-seeking. Communication and social skills were enhanced as children learned to respect each other:

His behaviour was more aggressive last term. He is calm, polite and respectful toward his friends now.

PA, no one would believe that he would sit down and draw for an hour in a peaceful way.

YB would take things without permission and refuse to share learning materials with others. Over time, he learned how to share learning tools with the group, seeks permission before he takes something from others, and learned how to apologise. For example, he took a paint pen from a classmate and this caused friction between them. Through acting play, he was shown how he could ask for the pen.

YZ become calmer and the class was more harmonious. At first, he said they didn’t want to be friends with Roma children in the class. He said, they can’t be my friends because they are gypsy… He gets on well with them…the group activities are more friendly and harmonious.

These children seemed to have experienced adverse childhood events or that they had insecure attachment relationship with primary figures which then made them less motivated to learn. From the above, the provision of attachment-based learning activities seemed to have motivated children to adapt better behaviour, creating a better school environment. They learned how to share class materials and reflected on past difficult behaviour. For example, *PA would sit down and draw for half an hour in a peaceful way; the group activities were more friendly and harmonious.* Vygotsky (1978, cited in White and Warfa, 2011) suggested that *learning precedes development and development is best facilitated by understanding a child’s zone of proximal development.* In the zone of proximal development (ZPD), caregivers are expected to help children cope with anxious and fearful situations. It is within this attachment-based proximal zone children can learn how to regulate stressful situations. In turn, this enhances the child’s learning development, emotional regulation and mental well-being.

### Children with non-improved behaviour

In contrast to the group of children who improved, limited or no improvements were observed for the following children:

It is difficult to talk about MY's progress because of absenteeism. He came to school after three months. He is still keen to learn new things but absenteeism is a huge obstacle. This makes impossible to make progress with him.

IY’s behaviour became more aggressive, compared with previous days.

This suggests that the psychosocial programme had little impact on the above group of children. We do not have a single explanatory factor, although there are several possibilities. Absenteeism has been a recurrent concern over the entire duration of the psychosocial intervention for this group. Those children with the least improved behaviour were observed to have been mostly absent from the school for long periods (See Table 2).

Chronic absenteeism seems to have limited these children’s opportunity to develop adequate social skills and psychological resilience from the carefully planned psychosocial activities. In some cases, it seems that children made improvements before their behaviour deteriorated following a long period of absence from the school. In the international literature, absenteeism is viewed as a form of educational neglect [26]. We observed that significant number of children would be absent from the school for any period from three to eleven weeks. Van Wert et al. [26] illustrates how absenteeism is often linked with *children’s internalising and externalising problems, family poverty, mental health issues, homelessness, substance abuse, crime and a lack of basic necessities* (See [26]: 50). Given that children attending this school came from marginalised and socially excluded communities with unmet basic needs including health, education, housing and employment needs. Absenteeism is one of the possible reasons for the lack of emotional and behavioural progress observed among the non-improved children:

ERS was too distracting in all activities. His disruptive behaviour destroyed group dynamics.

ERN liked games but her attention would fall apart quickly, difficult to keep her in game activities

RB was very emotional and intense. For example, when we were talking about simple rules, we had to be extra careful.

Frequent distractions, persistent disruptive behaviour, poor concentration skills and intense emotions may all be rooted in childhood adversities. These adverse childhood experiences hinder educational development and psychological wellbeing. Van Wert et al. [26] proposed that lack of pre-school attendance, poor prosocial skills and lack of parental warmth are risk factors for poor academic functioning. These are problematic constructs that may also explain why some children have not benefited from the eight-months-long psychosocial programme. To this end, children from dysfunctional households and with more serious form of insecure attachment behaviour may require longer psychosocial interventions. Longer duration and focused health and social care work would be suitable for such a non-improved group of children with various vulnerabilities and complex psychosocial needs.

## Discussion

We used a collaborative psychosocial programme to address concerns of deprived school children with issues of violence, bullying, emotional problems, disruptive behaviour, educational neglect, and other childhood adversities. The attachment theory proposes that children who are brought up in a secure and supportive environment are likely to develop an effective emotional regulation apparatus and strategies for coping with psychological instability and uncertainty throughout the different stages of the life course. From the psychosocial programme, significant number of children from year-one classes lacked social skills and those other soothing psychological and emotional resources that aid the learning process and developmental maturity.

At the start of the psychosocial intervention, we noted that violence and disruptive behaviour was out of control with children openly fighting and bullying each other in several school settings. Violence and bullying are well-researched risk factors for emotional instability and psychological problems. Children can develop complex trauma from abuse and violence exposed to them during the formation years and early childhood stages [15]. Traumatised children struggle in school settings as the anxieties and mistrust emerging from traumatic experiences and adverse childhood experiences grow with them. Also, children may resort to aggressive behaviour as a defensive mechanism or as a way of coping with psychological anxieties. Such children struggle with school rules and ordinary social interactions creating an environment in which quality teaching and effective learning activities are challenged. Felitti et al. [14] provides a comprehensive analysis into the relationship between exposure to abuse, household dysfunction and multiple risk factors for behavioural problems. Exposure to abuse and household dysfunctions were associated with adult morbidities and mortalities. All the data emerging from population studies underscore the importance of addressing childhood adversities and educational neglect as a matter of societal priority. Their study shows the implications of adverse childhood experiences for poor mental and physical health in adult life.

From a psychosocial perspective, we collaborated with school-teachers, school management, support staff, local municipality offices (education, health and social work), community groups, voluntary organisations, clinicians, researchers and parents to deliver support to school children with considerable adverse life adversities. For a period of 8 months, university psychology student mentors provided social and psychological support to struggling school children, in a sense acting as caregivers. Through educational and social activities (library visits, reading, writing, and listening games, painting games, maths games, handwashing games, drawing, animal animation, calendar making, leaf making, puzzle games, garden games, ball games, parachuting games, colouring, hula-hoop and driver-car role play), the psychology student mentors were able to help the pupils to regulate intense internal and external emotions. They provided a comprehensive psychosocial support to improve children’s basic psychological and social needs by creating a safe and secure environment in which disruptive behavioural problems, emotional sensitivities, interactivity, socialisation, empathic attachment and interpersonal communications (intrapsychic and interpersonal psychological processes) were addressed through a series of weekly actions and activities. The improvements noted from the weekly observations are supported by the existing attachment theory and psychosocial frameworks as well as the school-teachers’ perceptions of the outcomes of the psychosocial intervention. There were recurrent themes of children making significant emotional and behavioural improvements. Children with experiences of insecure and disorganised attachment behaviour begun enjoying the school environment. In turn, this improved their focus and concentration abilities. To this end, the findings of the study highlight the benefits attachment informed psychosocial programmes have in improving social, emotional and psychological well-being for children with educational neglect and multiple difficult life experiences. We carried out a separate study with school-teachers and support staff to cross-validate the psychology student mentors’ weekly observations, see Bademci et al. [3]:

They (University students) established such a good and gentle relationship with children that the children started to express more positively (Teacher, Female).

They played together more calmly without hurting each other. At least, they followed the university students’ examples (Teacher, Female).

It affected pupils’ sense of self-worth. They feel safe. The feel worthy. These children don’t receive appraisals neither from families nor from the streets (Teacher-parent Associate)

The children are happier when the university students come. An absentee student would also attend if their friend was in the class (Teacher, Male)

From the teachers’ perspectives, the weekly psychosocial intervention reduced disruptive behavioural problems and insecure attachment anxieties whilst strengthening self-confidence, self-worth and social skills. The Attachment Aware School (AAS) framework [22] suggests a pyramid model for responding to the needs of school children with insecure attachment behaviour. At the top of the pyramid are children with highest needs. This group of children would benefit from specialist psychological services. In the middle are children with medium unmet needs who would benefit from a tailored support programme. At the base of the pyramid are the largest number of children with no or low level of unmet needs. We implemented an attachment-based psychosocial programme designed to respond to the needs of those with unmet emotional, social, and psychological needs (those at the middle of the pyramid). They received a holistic psychosocial intervention through various learning activities and role-model relationships. At the end of the psychosocial programme, improvements were made in several areas. For some children, their self-confidence increased whilst aggressive behaviour decreased. Others acquired new social and interpersonal skills.

## Conclusion

Every child needs to have access to inclusive, equitable, supportive, and effective education to gain sustainable and meaningful life skills. Crucial psychosocial skills and emotional stability are necessary for successful later adult life. Investment in children’s education and wellbeing is an effective long-term strategy for sustainable economic growth, stable and just society. Various research studies report the consequences of educational neglect and the adverse multiple costs to individuals, families, communities and society-at-large. In middle income countries like Turkey, significant number of children remain out of school or leave school early without adequate education (see [2]). Children excluded from school education face more violence and abuse than other groups. Children excluded from early learning are likely to live in a circle of abject poverty and have poorer physical and mental health status. To break the inter-generational cycle of child poverty, child abuse and marginalization, there is a need for ‘flexible’ multidisciplinary interventions for understanding and responding to the complex impacts educational neglect has on the social and psychological wellbeing of children. While most developed countries provide access to social work and mental health services to children with educational neglect and difficult psychological experiences, support in low and middle-income countries is restricted. Moreover, school roles in many of these countries are limited to curriculum delivery and academic activities. We call for a coordinated action involving core stakeholders (research councils, local municipality offices, schools, universities and higher education institutes and community organisations; to mention a few) to organise access to inclusive education for neglected children. This is what the psychosocial model is about.

Finally, the work we presented here has several strengths and limitations. We examined the evolving application of the attachment theory and links to psychosocial models. We implemented an attachment informed psychosocial programme for a vulnerable group of children with one group of children making improvements in several emotional and behavioural areas. While the flexibility and collaborative nature of the social action research allowed us to successfully implement an attachment-informed psychosocial programme, the findings presented in this paper cannot be generalised beyond the reference group as this is in line with qualitative designs. McWilliam and colleagues [17] provided in-depth analysis on how knowledge translators and implementation scientists struggle with generating evidence-based research through social action research and social interactions. This is partly because such social interactions compound with ever changing *dynamics and subjectivity* which then makes the generalization of results derived from qualitative methods challenging to apply beyond the reference group. Another issue is to ask the extent to which the psychosocial programme activities would have made more positive impact (particularly for children with high levels of unmet needs) if the intervention was more clinical or specialised. The answer is probably yes, (for example, see [28]). Turkey is a middle-income developing country and social scientist researchers are often confronted with limited funding opportunities. Within this context, we devised an innovative weekly psychosocial programme to empower a local elementary school children with emotional and behavioural issues. Our plan for future research is to secure funding which will allow us to carry on with the provision of therapeutically informed psychosocial interventions for children from deprived communities with considerable unmet psychosocial needs.

## Data Availability

This paper contains no quantitative data. Inquiries about the qualitative materials used in the manuscript can be made to the corresponding author.
